# Biofluid-Derived Exosomal LncRNAs: Their Potential in Obesity and Related Comorbidities

**DOI:** 10.3390/biology13120976

**Published:** 2024-11-26

**Authors:** Ebenezeri Erasto Ngowi, Tuyan Lu, Qing Liu, Xianghong Xie, Ning Wang, Liping Luo, Lijuan Deng, Yinghua Zhou, Zhihong Zhang, Aijun Qiao

**Affiliations:** 1 Zhongshan Institute for Drug Discovery, Shanghai Institute of Materia Medica, Chinese Academy of Sciences, Zhongshan 528400, China; ebenezerngowi92@gmail.com (E.E.N.); lutuyan966@zidd.ac.cn (T.L.); liuqing334@zidd.ac.cn (Q.L.); xiexianghong0578@zidd.ac.cn (X.X.); wangning@zidd.ac.cn (N.W.); luoliping924@zidd.ac.cn (L.L.); denglijuan538@zidd.ac.cn (L.D.) zhouyinghua0342@zidd.ac.cn (Y.Z.); zhangzhihong0566@zidd.ac.cn (Z.Z.); 2 Shanghai Institute of Materia Medica, Chinese Academy of Sciences, 555 Zu Chong Zhi Road, Shanghai 201203, China; 3 University of Chinese Academy of Sciences, Beijing 101408, China

**Keywords:** biofluids, exosomes, long non-coding RNAs, obesity and associated diseases

## Abstract

Obesity has escalated into a critical global health crisis, tripling in prevalence since the mid-1970s. This increase mirrors the rise in metabolic-associated diseases such as type 2 diabetes (T2D) and its complications, certain cancers, and cardiovascular conditions. While substantial research efforts have enriched our understanding and led to the development of innovative management strategies for these diseases, the suboptimal response rates of existing therapies remain a major obstacle to effectively managing obesity and its associated conditions. Over the years, inter-organ communication (IOC) has emerged as a crucial factor in the development and progression of metabolic disorders. Exosomes, which are nano-sized vesicular couriers released by cells, play a significant role in this communication by transporting proteins, lipids, and nucleic acids across cellular landscapes. The available evidence indicates that exosomal RNAs present in biofluids such as blood, urine, milk, vitreous humor (VH), and cerebrospinal fluid (CSF) are altered in numerous diseases, suggesting their diagnostic and therapeutic potential. Long non-coding RNAs contained in exosomes (exo-lncRNAs) have attracted considerable interest, owing to their ability to interact with critical components involved in a multitude of metabolic pathways. Recent studies have found that alterations in exo-lncRNAs in biofluids correlate with several metabolic parameters in patients with metabolic-associated conditions; however, their exact roles remain largely unclear. This review highlights the diagnostic and therapeutic potential of exosomal lncRNAs in obesity and its associated conditions, emphasizing their role in IOC and disease progression, aiming to pave the way for further research in this promising domain.

## Simple Summary

Exosomal cargo serves as a crucial mediator of cell-to-cell communication. The evidence suggests that exosomal long non-coding (exo-lncRNAs) are altered in the biofluids (such as blood, vitreous humor, and tears) of patients with metabolic disorders and correlate with key metabolic parameters, including body mass index, inflammation, and insulin sensitivity. This indicates their potential as biomarkers for monitoring and managing obesity and related metabolic diseases. This review emphasizes the diagnostic and therapeutic potential of exo-lncRNAs in obesity and related comorbidities, highlighting the need for further investigation in this promising area.

## 1. Introduction

Obesity has escalated into a critical global health crisis, tripling in prevalence since mid-1970s and currently impacting over 1 billion people, including nearly 880 million adults and approximately 160 million children and adolescents aged 5–19 [1]. This rise is fueled by factors such as genetics, sedentary lifestyles, poor diets, and environmental aspects. The prevalence of obesity is linked to an increase in both metabolic and non-metabolic diseases including diabetes, heart disease, and cancer [2,3]. Current obesity management programs adopt a multidisciplinary approach, integrating lifestyle modifications, behavioral therapy, and, when necessary, pharmacotherapy and surgery. In light of the escalating prevalence of obesity and the inadequate efficacy of current treatments, the advent of novel therapeutics becomes imperative. A promising strategy involves leveraging the complex parameters altered by obesity and its metabolic conditions to identify effective therapeutic targets. Notably, inter-organ communication (IOC) harbors a crucial insight, enabling a deeper understanding of how various secreted elements from different organs influence pathophysiology. IOC is facilitated by both the circulatory system (including secretory factors and small extracellular vesicles (sEVs)) and the nervous system (through neurotransmitters such as epinephrine) [4,5].

A prominent example of IOC is the gut–brain axis, which links the gastrointestinal tract to the central nervous system and influencing brain function [6]. Other notable IOC networks include the pancreas’s interaction with the liver, adipose tissue, and muscle via insulin secretion, as well as the signaling of adipokines from adipose tissues to the liver and brain [7], and hepatokines, insulin-like growth factor 1, and angiotensinogen, which regulate growth, metabolism, and blood pressure [8]. A recent study has identified activin E as a novel factor released from the liver in response to elevated free fatty acids and triglycerides; it sends signals through activin receptor-like kinase 7 in adipose tissue to suppress lipolysis, thereby reducing fatty acid flux to the liver and preventing excessive hepatic lipid accumulation [9]. In addition to these secreted factors, recent research developments have shone a spotlight on sEVs and their cargoes in relation to IOC, revealing their potential for the diagnosis and treatment of several diseases [10,11]. Consequently, analyzing the contents of biofluids holds immense potential for uncovering valuable insights into various diseases, as well as their diagnostic and prognostic feasibility.

Exosomes are nano-sized membranous sEVs that play a pivotal role in facilitating cellular communication through their specific surface proteins and diverse cargo, encompassing proteins, lipids, and nucleic acids [12]. Exosomal RNAs are increasingly recognized as key players facilitating IOC. Among the different types of ribonucleic acids (RNAs) present in exosomes, long non-coding RNAs (lncRNAs) have garnered substantial interest due to their critical regulatory roles in various biological processes and their associations with numerous diseases [13,14,15]. LncRNAs are transcripts that exceed 200 nucleotides in length and do not encode proteins; instead, they serve as important regulators of gene expression by often acting as scaffolds or decoys for proteins and microRNAs (miRNAs) [16,17,18]. The potential of exosomal long non-coding RNAs (exo-lncRNAs) in biofluids has been primarily and extensively studied in the context of cancer [19,20,21]. However, recent years have witnessed a growing interest in investigating exo-lncRNAs in obesity and related conditions [22,23].

In metabolic diseases, epigenetic alterations have been shown to affect the expression of various lncRNAs, potentially contributing to their pathogenesis [24,25,26,27,28]. Similarly, accumulating evidence indicates that exo-lncRNAs in biofluids are significantly altered and correlate with various metabolic parameters [29,30,31,32], positioning them as promising diagnostic and therapeutic candidates. The construction of interaction networks, coupled with loss- and gain-of-function assays, have further confirmed that exo-lncRNAs can target critical signaling pathways involved in regulating nutrient metabolism, inflammation, apoptosis, and oxidative stress, processes that are often disrupted in metabolic-related conditions. Despite the increasing recognition of exo-lncRNAs in these conditions, this field remains underexplored, particularly regarding biofluids beyond blood, the origin and destination of exo-lncRNAs in these biofluids, and the identification and classification of novel lncRNA transcripts. In addition, much work remains to be conducted regarding the underlying mechanisms and precise effects mediated by these lncRNAs.

So far, several reviews have emphasized the potential of circulating non-coding RNAs, including exo-lncRNAs, in the context of metabolic diseases, especially those detectable in blood [24,33,34,35]. Therefore, this review focuses exclusively on exo-lncRNAs, expanding the scope to include other biofluids, particularly those from bodily cavities. By addressing this knowledge gap, we aim to provide a comprehensive exploration of exo-lncRNAs and set the stage for future discoveries that could revolutionize the diagnosis and treatment of metabolic-related diseases.

## 2. LncRNAs Sorting into Exosomes

The biogenesis of exosomes through both endosomal sorting complex required for transport (ESCRT)-dependent and -independent pathways has been extensively reviewed in previous studies [36,37,38]. Therefore, this chapter will focus on the incorporation of lncRNAs into exosomes. The cellular milieu significantly influences the selection and incorporation of lncRNAs into exosomes, leading to the generation of exosomes that exhibit distinct lncRNA profiles shaped by cellular conditions. The sphingomyelin cycle, an alternative pathway to ESCRT, is essential in the biogenesis of ceramide-enriched exosomes and cargo sorting [39]. In this cycle, sphingomyelin is converted by sphingomyelinases into ceramide and phosphorylcholine, with ceramide further metabolized into sphingosine and sphingosine-1-phosphate [40,41]. Targeting this pathway through gene silencing or the neutral sphingomyelinase-2 inhibitor GW4869 can significantly disrupt exosomal biogenesis, cargo packaging, and release [42]. Saliently, according to Kong et al., treating human adipose-derived stem cells with GW4869 can also decrease the levels of the exo-lncRNA MALAT-1, while the stimulation of ceramide synthesis using C2- and C6-short-chain ceramides results in its upregulation [43], indicating its involvement in packaging lncRNAs.

**Figure 1:**
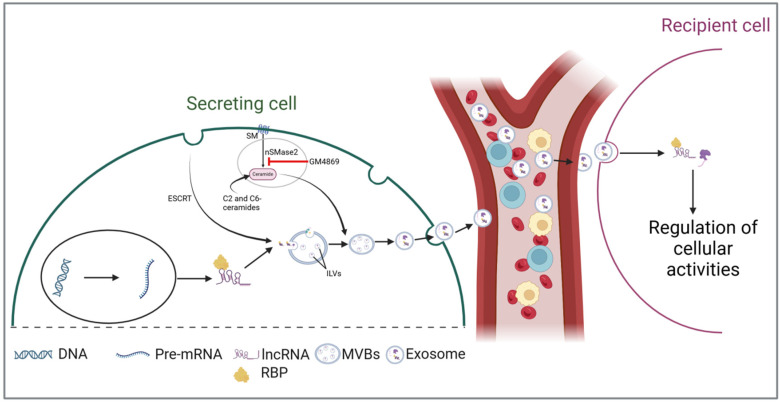
LncRNAs are secreted and incorporated into multivesicular bodies (MVBs) with the assistance of RNA-binding proteins (RBPs). LncRNAs are incorporated into multivesicular bodies (MVBs) with the assistance of RNA-binding proteins (RBPs). During the maturation of early endosomes into MVBs, these lncRNAs are sorted into intraluminal vesicles (ILVs) through the endosomal sorting complex required for transport (ESCRT)-dependent and -independent pathways. During exosomal secretion, ILVs are released as exosomes into biofluids, facilitating their transport to target cells and enabling intercellular communication. (Abbreviations: intraluminal vesicles (ILVs); multivesicular bodies (MVBs); RNA-binding proteins (RBPs); endosomal sorting complex required for transport (ESCRT); sphingomyelin (SM); neutral sphingomyelinase-2 (nSMase2)).

Although the complete sequence of events through which lncRNAs are loaded into exosomes is not yet fully understood, the existing literature underscores the critical role of RNA-binding proteins (RBPs) in this intricate process (Figure 1). RBPs are crucial regulators of RNA metabolism, playing a significant role in RNA transport by forming ribonucleoprotein complexes [44,45]. These complexes facilitate the transport of various RNA species into exosomes during biosynthesis [46,47]. Statello et al. demonstrated that silencing specific RBPs, particularly the major vault protein (MVP), significantly reduces total RNA levels within exosomes [48]. Conversely, the overexpression of MVP increases RNA loading. Another RBP, heterogeneous nuclear ribonucleoprotein A2B1 (hnRNPA2B1), has also been shown to facilitate RNA packaging into exosomes by binding to the GGAG motif [49]. By using RNA immunoprecipitation and RNA pull-down assays, Lei et al. confirmed the interaction between hnRNPA2B1 and the GGAG motif located at the 5′end of lncRNA H19 [50]. The authors further revealed that mutations in this motif or silencing of hnRNPA2B1 significantly hindered both the interaction and the loading process. A similar mechanism has been reported for other lncRNAs, including lncRNA LNMAT2 [51] and AGAP2-AS1 [52]. Together, the above evidence implicates that RBPs are crucial in the packaging of lncRNAs into exosomes. However, additional research is needed to uncover other potential mechanisms that may be involved.

## 3. Exosomes in Biofluids

**Figure 2:**
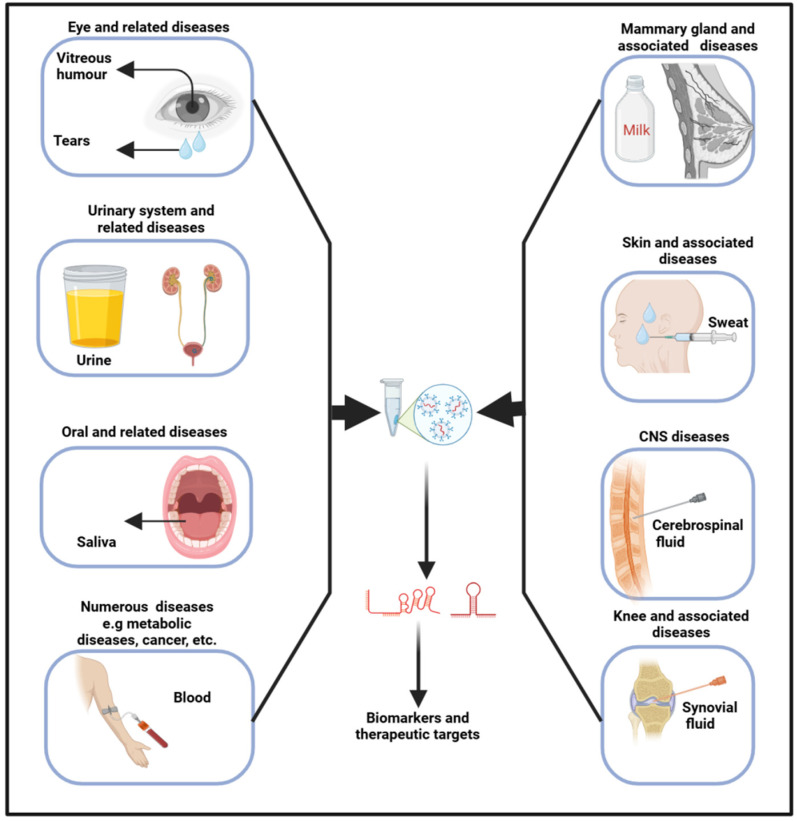
Exosomes obtained from biofluids hold significant promise as biomarkers for diagnosing a range of diseases. Specifically, exosomal lncRNAs present in these fluids can serve as indicators for various pathophysiological conditions.

Exosomes originate from the inward budding of the endosomal membrane, leading to the genesis of multivesicular bodies [53]. Upon merging with the plasma membrane, exosomes are subsequently released into the extracellular milieu, where they are transported by biofluids into recipient cells. As a result, biofluids, encompassing any liquid (such as interstitial fluid, saliva, tears, milk, urine, sweat, blood, VH, CSF, and so on) within an organism, have emerged as paramount in diagnosing and treating various diseases, including neurodegenerative [54], osteoporosis [55], and cancers [55,56]. In biofluids, several contents, including RNAs and proteins, can be found in cells (blood cells), lipids (high-density lipoproteins), and vesicles (exosomes) [57]. The release of exosomes, along with their size, content, and function, exhibits significant variability under both physiological and pathological conditions, thereby underscoring their considerable diagnostic potential [58]. These fluids harbor a multitude of entities and molecules, each brimming with profound theranostic potential (Figure 2). As their pivotal role continues to unfold, they are now shaping new frontiers in medicine, heralding a new era of precision medicine.

Liquid biopsy, a sophisticated method that harnesses biofluids, provides a non- to less-invasive approach for detecting and monitoring disease biomarkers, thereby significantly enhancing diagnostic accuracy and optimizing patient outcomes. The technique has demonstrated that exosomal cargo can be utilized for both the diagnosis and prognosis of diseases [56,59,60]. Wu et al. characterized sweat exosomes and identified, among others, 14 antimicrobial peptides, suggesting these vesicles play a role in immune regulation [61]. Similarly, the analysis of tear exosomes has unveiled several proteins and miRNAs that have potential utility in diagnosing eye diseases and associated conditions [62]. Moreover, a study by Xiao et al. found that the plasma exosomes of acute myeloid leukemia patients are low in LINC00265, LINC00467, and UCA1 and are high in SNHG1 when compared to the controls [63]. Indeed, their combined analysis demonstrated superior diagnostic efficiency. In summary, exosomal cargoes are pivotal in regulating cellular activities; hence, their analysis in biofluids could therefore yield invaluable insights into the physiological state of the body, enhancing our understanding of its complex, dynamic processes. The area holds substantial potential for prognosis prediction; despite this, the specific targets are still under investigation.

## 4. Mechanisms of Action for Exosomal lncRNAs

Exo-lncRNAs can potently influence the expression and activity of mRNAs and proteins through a range of mechanisms, thereby enhancing their functions. One such mechanism involves serving as competing endogenous RNAs (ceRNAs). It is widely recognized that lncRNAs competitively bind to miRNAs, restricting miRNA–mRNA interactions, and ultimately influence cellular processes [64,65,66]. In addition, other studies have revealed that lncRNA can also exert their effect by directly binding to mRNAs [67,68,69]. Furthermore, exo-lncRNAs have been identified as capable of interacting with proteins [70,71,72].

Regarding metabolic parameters, several studies utilizing network prediction analyses have identified that exo-lncRNAs can function as ceRNAs for key genes associated with metabolism [26,73]. In vitro assays have shown that lncRNA H19 interacts with miR-467, while MIAT binds to miR-133a-3p to exert their effects on metabolic pathways [74,75]. Similarly, lncRNA MALAT1 can bind to miR-382-3p and mediate the downstream regulation of cell proliferation and apoptosis in a diabetic model [76]. Moreover, high expressions of exo-lncRNA-p3134 promote the mRNA expression of transcription factor 7-like 2 in Min6 cell lines and mouse islet cells, contributing to insulin resistance [77]. However, it remains unclear whether lncRNA-p3134 directly binds to this factor or acts as a ceRNA to modulate its effects.

**Figure 3:**
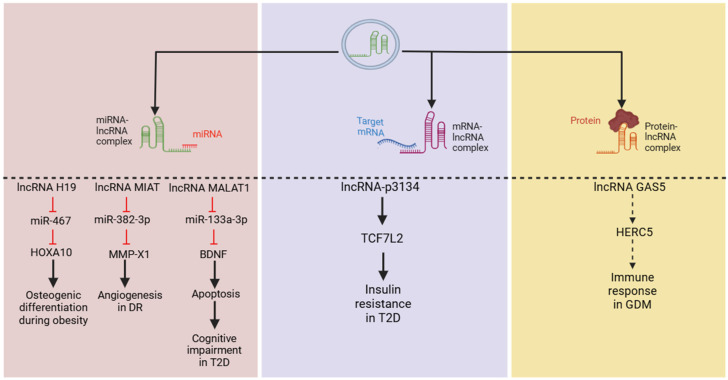
LncRNAs employ various mechanisms to regulate cellular activities, including targeting microRNAs (miRNAs), messenger RNAs (mRNAs), and proteins, all of which have been reported in metabolic diseases. From left to right, lncRNAs H19, MIAT, and MALAT1 have been shown to act through a classical pathway by binding to miRNAs to regulate their downstream targets. Additionally, lncRNA-p3134 may upregulate TCF7L2 (mechanism not clear) to mediate insulin sensitivity, while GAS5 has been predicted to dock with the HERC5 protein to induce GDM. (Abbreviations: long non-coding RNA (lncRNA); transcription factor 7-like 2 (TCF7L2); HECT and RLD domain-containing E3 ubiquitin protein ligase 5 (HERC5); gestational diabetes mellitus (GDM); diabetic retinopathy (DR); type 2 diabetes (T2D).)

Furthermore, a recent study on gestational diabetes mellitus (GDM) provided evidence of exo-lncRNA–protein interactions, demonstrating that lncRNA GAS5 can dock with HECT and RLD domain-containing E3 ubiquitin protein ligase 5 (HERC5), a critical regulator of immune response [78,79], suggesting lncRNA–protein interactions. Overall, these findings suggest that exo-lncRNAs can regulate various cellular processes and significantly influence cellular activities (Figure 3). However, further research is needed to elucidate their precise underlying mechanisms through in vitro and in vivo studies.

## 5. Circulating Exosomal lncRNAs in Metabolic-Associated Comorbidities

### 5.1. Obesity

The tide in the global obesity landscape has ignited an urgent call to action, spurring a race to discover innovative therapeutic targets and opening doors to transformative solutions for this epidemic. In response, emerging research frontiers have unveiled innovative strategies poised to redefine treatment options through novel approaches. Notably, one such strategy involves harnessing exo-lncRNAs to regulate metabolic parameters, potentially unlocking a treasure trove of groundbreaking treatment modalities.

A burgeoning corpus of evidence delineates distinct alterations in circulating lncRNAs in obese individuals when juxtaposed with those of normal weight [26,80]. A prior study elucidated that myriad circulating lncRNAs are deregulated in obesity, identifying markedly reduced levels of lncRNA-p5549, -p21015, and -p19461 in obese individuals compared to their non-obese counterparts [32]. The aforementioned lncRNAs display a negative correlation with obesity metrics, encompassing body mass index (BMI), waist circumference, and insulin resistance. A microarray analysis of insulin-resistant mice on a high-fat diet (HFD) unveiled a staggering 375 differentially expressed (DE) lncRNAs in serum exosomes, with the most significant ones closely linked to essential metabolic functions such as fatty acid metabolism, glycerolipid metabolism, and protein processing [81]. Additionally, exo-lncRNA HOTAIR levels are substantially higher in the blood of obese individuals compared to lean counterparts, with this increase further exacerbated by a sedentary lifestyle [30].

Furthermore, common strategies for managing obesity include weight loss programs such as dietary interventions and physical activity can significantly influence exo-lncRNAs to exert their effects. For instance, exercise has been shown to elevate the expression of several plasma exo-lncRNAs, including obesity-associated lncRNA GRNDE, in both human and mouse models [31,82,83]. Similarly, a 12-week diet-induced weight loss program has been shown to reverse the downregulation of the obesity-associated lncRNA p19461, suggesting its potential as a biomarker for successful weight loss and improved insulin sensitivity [32].

Adherence to mediterranean-based diets (MDs) has also been closely associated with improved weight loss and metabolic parameters [84,85]. Ergo, a study utilized the plasma exosomal data collected from 150 T2D-free participants (BMI = 25–35 kg/m^2^) at baseline and after one year on either a low-fat diet or MD enriched with extra-virgin olive oil (EVOO) or nuts (MD + Nuts) to determine the impact of MDs [86]. They found that, compared to the low-fat diet, MD + EVOO had 413 altered lncRNAs while MD + Nuts had 476. The common signaling pathways targeted include PI3K-Akt and AMPK in the low-fat diet; PI3K-Akt, NF-kappa B, HIF-1, and insulin resistance in MD + EVOO and FoxO; and PI3K-Akt, AMPK, p53, and HIF-1 in MD + Nuts. While current obesity diagnosis methods are adequate, the above evidence succinctly suggests that utilizing exo-lncRNA profiling can provide deeper insights into an individual’s metabolic state, identifying not only obesity but also other coexisting conditions. Also, they can enhance patient monitoring, facilitate personalized treatment approaches, and allow for the assessment of the body’s response to treatment.

### 5.2. Metabolic Syndrome (MetS)

MetS, hallmarked by dyslipidemia, inflammation, and pervasive insulin resistance, underpins the pathogenesis of diabetes and CVDs [87]. Obesity, particularly abdominal and visceral obesity, is a pivotal determinant in the pathogenesis of MetS [88]. MetS diagnosis necessitates the presence of at least three of the following criteria: hypertension, elevated fasting glucose levels, increased waist circumference, diminished HDL cholesterol, and elevated triglycerides. Recent research has identified a substantial number of circulating exo-lncRNAs altered in MetS. The analysis of systemic blood has explicitly uncovered 191 lncRNAs exhibiting differential expression between individuals with MetS and healthy controls [26]. Following the construction of a regulatory network, the authors demonstrated that these lncRNAs could potentially function as ceRNAs, interconnecting 13 lncRNAs to 8 miRNAs and 64 mRNAs. The key lncRNA regulators identified in this network were NR2F1-AS1, PART1, FOXC2-AS1, and PSMA3-AS1. Together, this compelling connection implies that circulating exo-lncRNAs may play a pivotal role in the etiology of obesity, although further studies are needed to determine their exact mechanisms of action.

### 5.3. Knee Osteoarthritis (KOA)

KOA is a prevalent degenerative joint disease marked by the progressive deterioration of articular cartilage, resulting in pain, stiffness, and functional impairment, significantly impacting the quality of life for affected individuals [89]. Obesity stands as a formidable risk factor for KOA; thus, rising obesity rates may exacerbate the incidence of KOA cases. Higher BMI correlates with a greater likelihood of developing KOA, particularly in individuals with a BMI of over 30 kg/m^2^ [90,91]. Wu et al. identified 196 lncRNAs altered in KOA individuals compared to controls [92]. Notably, synovial-fluid-derived exo-lncRNA PCGEM1 has been reported to be significantly dysregulated in a stage-dependent manner in KOA patients [93]. In relation to obesity, one study reported an altered plasma exo-lncRNA profile in obese patients with KOA compared to non-obese KOA patients, identifying 29 DE lncRNAs [94]. Among these, seven lncRNAs, including TAL1-3-2, NONHSAT209148.1, DLEU2, LINC00969, CABP4-2, CHD1L-5, and ERICH1-19 were potently linked to lipid metabolism. These findings highlight the complex relationship between obesity and KOA, suggesting a significant role for exo-lncRNAs in this interplay.

### 5.4. Obstructive Sleep Apnea (OSA)

Obesity is the leading risk factor for developing OSA, a condition marked by repetitive upper airway obstruction during sleep [95,96]. This disorder is closely linked to a variety of cardiovascular diseases, including hypertension, coronary artery disease, and heart failure. The plasma exosomal expression of lncRNA ENST00000592016 has been reported to be elevated in OSA patients compared to controls [73]. This increase in expression positively correlates with critical metrics for evaluating OSA, including the apnea–hypopnea index and oxygen desaturation index, as well as with the severity of OSA and BMI. Furthermore, network analysis indicates that this lncRNA may target critical signaling pathways, including the PI3K-Akt, MAPK, and TNF pathways, suggesting its potential involvement in the underlying mechanisms of OSA.

### 5.5. Osteoporosis (OP)

OP is a bone condition characterized by the deterioration of bone density and structure, leading to increased fragility and a greater susceptibility to fractures [97]. This condition is most prevalent among the elderly. The relationship between obesity and OP remains contentious. Earlier evidence suggested obesity could be beneficial for OP [98]. This correlation primarily stems from the fact that bone mineral density typically increases with higher BMI [99]. However, this connection may not necessarily confer benefits for osteoporosis management. In fact, subsequent studies that accounted for the mechanical loading effect of body weight on bone mass have suggested otherwise [100,101,102]. Furthermore, analyses of the relationship between BMI and fractures at various skeletal sites indicate that, in older men, obesity is linked to a reduced risk of fractures in the clinical spine, hip, pelvis, and wrist/forearm, as well as an augmented risk of multiple rib fractures compared to men with normal or lower weight [103,104]. Nonetheless, circulating levels of lncRNAs, including GAS5, is elevated in the serum of osteoporotic patients and correlate with BMI [105]. Using both in vitro and in vivo methodologies, Wang et al. elucidated the critical role of lncRNA H19 in the compromised healing of fractures associated with obesity [74]. The authors found that obesity markedly reduces the expressions of lncRNA H19 in plasma and bone marrow mesenchymal stromal cell (BMSC) exosomes, thereby disrupting osteogenesis and impairing fracture healing. Expectedly, treatment with exosomes from normal BMSCs overexpressing lncRNA H19 restored its levels and enhanced the healing process in mice by competitively binding to miR-467, thus mitigating its suppressive effect on homeobox A10. This evidence suggests that exo-lncRNAs hold promise as diagnostic and therapeutic tools for osteoporosis, though this area warrants further exploration.

### 5.6. Type 2 Diabetes (T2D)

The dynamic relationship between obesity and T2D is compelling, with obesity acting as a powerful trigger that elicits the onset of T2D and accelerates the progression of this chronic disease. T2D has also been intricately linked to circulating lncRNAs. In particular, the lncRNAs MEG3 and TUG1 have been reported to be upregulated in circulating peripheral blood [106]. Similarly, lncRNA-p3134 is significantly upregulated fourfold in serum exosomes from diabetic patients and positively correlated with fasting blood glucose and insulin resistance markers [77]. Further analysis linked lncRNA-p3134 with the regulation of glucose homeostasis and β-cell function by suppressing apoptosis and glucotoxicity through the modulation of the PI3K signaling pathway.

Additionally, a study involving Mexican subjects identified MALAT1 and H19 as altered circulating serum exo-lncRNAs in patients with T2D [107]. Specifically, MALAT1 expression was significantly reduced in both serum and serum exosomes from T2D patients, whereas H19 levels increased, particularly among individuals with poor glycemic control, and showed a strong correlation with waist circumference. Furthermore, in the serum exosomes of T2D mice, lncRNA MALAT1 was found to be expressed at low levels, while its target miR-382-3p was markedly amplified [76]. Consequently, either silencing MALAT1 or overexpressing miR-382-3p in neuronal cells could reduce the expression of crucial metabolic markers such as insulin receptor substrates 1 and 2 as well the PI3K/AKT and Ras/MAPK signaling pathways. Moreover, aerobic exercise has been shown to notably elevate the serum levels of exo-lncRNA MALAT-1, subsequently suppressing miR-382-3p and enriching brain-derived neurotrophic factor expression, ultimately leading to improved metabolic indices [76,108,109]. In summary, the aforementioned findings indicate that circulating exo-lncRNAs are altered in T2D and could play a significant role in its pathogenesis by targeting essential signaling cascades.

### 5.7. Diabetic Retinopathy (DR)

DR is a key complication of T2D that can lead to severe vision impairment. The interplay between obesity, T2D, and DR is a captivating puzzle filled with controversies. While obesity and type T2D are closely linked, the connection between obesity and DR remains belligerent [110]. Nevertheless, lncRNAs have a vital role in regulating DR [111,112]. A previous study indicated that over 300 lncRNAs are DE in the retinas of early DR mice [113]. Additionally, elevated levels of lncRNAs ANRIL, H19, HOTAIR, HULC, MIAT, WISPER, and ZFAS1 were detected in the serum of diabetic patients across various stages of DR [114]. In plasma exosomes, several lncRNAs including DLX6-AS1, PRINS, and FAM190A-3 are DE in patients with T2D and those with DR [115]. Among these, the lncRNA DLX6-AS1 was found to be significantly upregulated in DR patients, while the lncRNAs PRINS and FAM190A-3 were downregulated. By utilizing logistic regression and receiver operating characteristic curve analyses, it was further demonstrated that a combination of the lncRNAs DLX6-AS1 and PRINS offers strong predictive capability for diagnosing the condition.

Furthermore, a novel lncRNA, LOC100132249, is enriched in exosomes from the vitreous humor (VH) of patients with proliferative DR [116]. Further analysis revealed that this lncRNA functions as a ceRNA for miRNA-199a-5p, thereby activating the Wnt/β-catenin pathway, subsequently modulating the SNAI1 promoter and ultimately endothelial dysfunction. The lncRNA MIAT is markedly elevated in VH exosomes from patients with proliferative DR and is involved in regulating the pro-angiogenic marker MMP-X1 by sequestering miR-133a-3p [75]. Collectively, these findings indicate that circulating exo-lncRNAs are essential for diagnosis and possess considerable therapeutic significance in DR.

### 5.8. Gestational Diabetes Mellitus (GDM)

Maternal obesity exerts a considerable influence on the onset of GDM [117]. The levels of lncRNA GAS5 in peripheral blood exosomes are significantly lower in pregnant women with GDM compared to those in the control group [78]. Using molecular docking, the authors further demonstrated that GAS5 may regulate immune response by binding to the ubiquitin ligase HERC5 protein. A study found significant differences in the expression of 256 lncRNAs in umbilical cord blood (UCB) exosomes between patients with GDM and healthy controls, with most of these exo-lncRNAs reported to contain miRNA binding sites [118]. Additionally, another study identified 372 DE lncRNAs in UCB exosomes together with numerous altered pathways including JAK/STAT, mTOR, PI3K/Akt, and TGF-β [119], suggesting that these exo-lncRNAs could be transported to the fetus and influence its growth and development.

## 6. Theranostic Potential, Challenges, and Perspectives

Exosomal RNAs found in biofluids exhibit notable changes across a range of pathophysiological conditions. These alterations are associated with the regulation of cellular functions and the coordination of responses to physiological stimuli, positioning them as key targets with substantial therapeutic potential. Specifically, exo-lncRNAs in biofluids show promise as non-invasive biomarkers for assessing and treating the pathophysiological conditions of various diseases [64,120]. In relation to metabolic diseases, the expression patterns of various exo-lncRNAs align with pivotal metabolic parameters, underscoring their potential as both biomarkers and therapeutic targets for metabolic-associated diseases, including KOA, OSA, MetS, OP, T2D, GDM, and DR (Table 1).

Interestingly, consistent findings have been reported across multiple conditions regarding the lncRNA HOTAIR. Specifically, elevated levels of HOTAIR have been observed in obesity, T2D, and DR, highlighting its potential as a valuable diagnostic and therapeutic target. This pattern also positions HOTAIR as a predictor for identifying individuals at higher risk, enabling timely interventions. Conversely, lncRNA H19 shows inconsistent findings; it is downregulated in obesity but upregulated in T2D and DR. While the levels of this lncRNA could provide insights into metabolic status and monitor disease progression, its therapeutic value is limited, making it more suitable for personalized medicine approaches. Overall, these findings underscore the complex regulatory networks involved in these diseases and highlight the need for further research into their underlying mechanisms.

Despite their considerable potential, there are a number of challenges that need to be thoughtfully surmounted in order to fully harness exo-lncRNAs as diagnostic and therapeutic tools. Across studies, exo-lncRNA quantification is complicated by high heterogeneity and lack of reproducibility across populations and experimental conditions. In essence, exosomes can vary significantly in size, content, and origin, which makes effective detection and consistent biomarker identification challenging. This discrepancy is caused by several factors, with the lack of standardized methods for isolating and characterizing exosomes being a significant contributor.

Additionally, many potential transcripts have yet to be classified as lncRNAs, hindering further research into their roles and functions. LncRNAs also possess numerous transcript variants, making it challenging to determine which variant has a stronger impact on a particular disease. To address these challenges, future research should involve large-scale cohort studies with standardized protocols for exosome isolation and analysis, along with advanced techniques for the rapid detection of low levels of exo-lncRNAs.

Another challenge is pinpointing the specific tissue or cells that contribute to the exo-lncRNAs present in biofluids. Although lncRNAs are typically known for their tissue-specific expressions [121], certain lncRNAs, such as H19, are altered in several tissues under obesity, including the liver, adipose tissue, muscle, and the heart under specific conditions [122,123,124,125,126], making it hard to confidently determine the contribution of each tissue to circulating exo-lncRNAs. To elucidate this, it is crucial for future studies to employ advanced techniques such as cell-specific metabolic labeling to confirm the origins and target organs of circulating exo-lncRNAs [127].

Obtaining biofluid samples in clinical practice involves various ethical considerations and requires patient consent, which can present significant challenges due to the inherent procedural risks involved. Although generally less invasive, certain biofluid collection procedures may involve puncturing vessels, canals, or cavities or may potentially cause pain, discomfort, or other adverse effects. Additionally, these procedures often demand specialized personnel and equipment, adding to the complexity. In some cases, the risks may outweigh the benefits, leading to a preference for more conservative diagnostic approaches. However, advancements in medical technology are progressively enhancing the feasibility and safety of biofluid collection, making it a promising avenue for future diagnostics.

In conclusion, exo-lncRNAs in biofluids represent a promising frontier in the quest for reliable biomarkers and diagnostic targets for metabolic-associated diseases. Their unique properties and roles in cellular communication suggest they could revolutionize our understanding and management of these complex conditions. To fully harness their potential, further research is essential to delineate their specific target organs, in vivo, as well as elucidate the intricate mechanisms through which they operate and examine their interactions within various metabolic pathways. With continued advancements in biomarker research and technology, exosomal lncRNAs hold the potential to revolutionize precision medicine for obesity and associated metabolic disorders.

## Figures and Tables

**Table 1:** Summary of potential lncRNAs linked to obesity and -associated diseases.

Disease	Model	Biofluid	Exosome Isolation Method	Quantification Method	Expression Profile vs. Control	Target(s) of Interest	Tissue of Origin	Target Tissue	Refs.
Obesity	Mice	Serum	Ultracentrifugation	Microarray and qPCR	Up: 285; Down: 90 lncRNAs	lncRNA AK018453	Adipose	Skeletal muscle	[81]
	Human	Serum	Ultracentrifugation	qPCR	Up: lncRNA HOTAIR	lncRNAs HOTAIR	Gluteal–femoral fat	Intestinal stem/progenitor cells	[30]
Metabolic syndrome	Human	Blood	exoRNeasy kit	RNA sequencing and qPCR	Altered 191 lncRNAs	lncRNAs NR2F1-AS1, PART1, -DLEU2, -PCA3, and -FOXC2	-	-	[26]
Knee osteoarthritis	Human	Plasma	Ultracentrifugation	qPCR	Up: lncRNA PCGEM1	lncRNAs PCGEM1	-	-	[93]
					Up: 15 lncRNAs; Down: 14 lncRNAs	lnc-TAL1-3-2, NONHSAT209148.1, lnc-DLEU2, Inc00969, lnc-CABP4-2, lnc-CHD1L-5, and lnc-ERICH1-19	-	-	[94]
Obstructive sleep apnea	Human	Plasma	Ultracentrifugation	RNA sequencing and qPCR	lncRNAs ENST00000442889, ENST00000592016, ENST0000561588, ENST0000662488, ENST0000594590, ENST00000567491, and ENST00000319701	lncRNAs ENST00000592016	-	-	[73]
Osteoporosis	Human	Plasma	Ultracentrifugation	qPCR	lncRNA H19	lncRNA H19	-	-	[74]
Type 2 Diabetes	Human	Serum	Ultracentrifugation	Microarray and qPCR	2269 lncRNAs	lncRNA-p3134	-	β-cells	[77]
	Human	Serum	exoRNeasy	qPCR	Up: H19; Down: MALAT1	lncRNAs H19 and MALAT1	-	-	[107]
Diabetic retinopathy	Human	Plasma	ExoQuick	RNA seq and qPCR	Up: DLX6-AS1; Down: lncRNAs PRINS and FAM190A-3	lncRNAs DLX6-AS1, PRINS, and FAM190A-3	-	-	[115]
	Human	Vitreous humor	Ribo Exosome Isolation solution	RNA seq and qPCR	Up: 547 lncRNAs; Down: 352 lncRNAs	lncRNA LOC100132249	Human retinal vascular endothelial cells	-	[116]
	Human	Vitreous humor	Ribo Exosome Isolation solution	qPCR	lncRNA-MIAT	lncRNA-MIAT	Human retinal vascular endothelial cells	-	[75]
Gestational diabetes mellitus	Human	Umbilical cord blood	Ultracentrifugation	Microarray and qPCR	372 lncRNAs; Up: lncRNAs AC006064.4 and lnc-HPS6-1:1; Down: lnc-ZFHX3-7:1	lncRNA AC006064.4	-	-	[119]

## Data Availability

Not applicable.

## Abbreviations

Long non-coding ribonucleic acid (lncRNA) type 2 diabetes (T2D); inter-organ communication (IOC); cerebrospinal fluid (CSF); exosomal long non-coding RNAs (exo-lncRNAs); small extracellular vesicles (sEVs); long non-coding RNAs (lncRNAs); exosomal lncRNAs (exo-lncRNAs); microRNAs (miRNAs); endosomal sorting complex required for transport (ESCRT); RNA-binding proteins (RBPs); major vault protein (MVP); heterogeneous nuclear ribonucleoprotein A2B1 (hnRNPA2B1); competing endogenous RNAs (ceRNAs); high-fat diet (HFD); differentially expressed (DE); mediterranean-based diet (MD); body mass index (BMI); metabolic syndrome (MetS); knee osteoarthritis (KOA); obstructive sleep apnea (OSA); osteoporosis (OP); bone marrow mesenchymal stromal cells (BMSCs); diabetic retinopathy (DR); gestational diabetes mellitus (GDM); HECT and RLD domain-containing E3 ubiquitin protein ligase 5 (HERC5).
